# Letter to the editor: will it be possible to achieve the global nutrition targets in Mexico by 2030?

**DOI:** 10.1186/s12939-024-02111-9

**Published:** 2024-03-18

**Authors:** Mónica Ancira-Moreno, Sonia Hernández-Cordero

**Affiliations:** 1 Departamento de Salud, Universidad Iberoamericana, Ciudad de México, México; 2 Observatorio Materno Infantil (OMI), Universidad Iberoamericana, Ciudad de México, México; 3 Instituto de Investigaciones para el Desarrollo con Equidad, Universidad Iberoamericana, Ciudad de México, México

## Abstract

In May 2012, the 65th World Health Assembly (WHA) approved six global nutrition targets by 2025 aimed to reduce stunting in children under five by 40%, maintain childhood wasting below 5%, halt obesity, cut anemia in women by 50%, lower low birth weight prevalence by 30%, and increase exclusive breastfeeding (EBF) within the first 6 months to 50%. These targets were extended to 2030, with all of them remaining as originally planned, but the EBF one (increased to 70%), wasting and overweight (both objectives set to eliminate them to negligible concern). Mexico is projected to achieve only one of the six nutrition targets (wasting) by 2025, falling far short of the stunting, low birth weight, anemia, and exclusive breastfeeding for the updated goals by 2030. This letter to the editor describes the most recent prevalence of malnutrition among mothers and children in Mexico. It discusses the challenges pregnant women and children under five years of age face exercising their right to good food, nutrition, and development. The authors reflect on the urgent need to make structural changes to achieve the global nutrition targets by 2030, highlighting the paramount importance of addressing the profound structural obstacles in Mexico and how Mexico’s government must prioritize poverty reduction, reduce the marked inequalities, enhance the quality of nutritional care and healthcare infrastructure, and implement climate-resilient agricultural practices to address this pressing issue.

Dear Editor,

In May 2012, the 65th World Health Assembly (WHA) approved a Comprehensive Implementation Plan on Maternal, Infant, and Young Child Nutrition, which outlined six global nutrition targets. These targets aimed to reduce 40% of the number of children under five who are stunted, reduce and maintain childhood wasting to less than 5%, halt the obesity epidemic, decrease 50% anemia in women of reproductive age, lower 30% the prevalence of low birth weight, and increase the rate of exclusive breastfeeding (EBF) in the first six months to 50% [1].

Subsequently, the nutrition targets were incorporated into the 2030 development agenda in its target 2.2: “end all forms of malnutrition.” In this sense, the World Health Organization (WHO) and the United Nations Children’s Fund (UNICEF) called for extending the targets to 2030 [2]. For anemia and low birth weight, targets remained unchanged based on a re-evaluation of trends, in line with the proposed 2025 target. The targets for EBF and stunting changed. For the former, it was aiming for a higher level of ambition to increase the rate from 50% to at least 70%. For stunting, the goal of reducing it by 40% n children under five years of age increased to 50%. As for wasting and overweight, the objective was set to eliminate them to negligible concern (prevalence less than 3%), which was deemed achievable. To attain these objectives by 2030, each nation must allocate resources according to its context and political and institutional preparedness to establish and expand relevant actions.

Taking 2012 as the baseline, the goals for Mexico by 2025 would be to achieve a prevalence of stunting of 8.1%, wasting of 1.6%, overweight and obesity of 9.7%, anemia in women of reproductive age of 5.8%, low birth weight of 4.9% and increase exclusive breastfeeding to 50%. In addition, according to the updated nutrition targets, by 2030, we would expect an increase in EBF rate up to at least 70% and a reduction in childhood wasting and overweight to less than 3%.

By 2012, when Global Nutrition Targets were endorsed, the prevalence of low birth weight in Mexico was 10.2%. For wasting, stunting, and overweight in children under five, it was 1.6%, 13.3%, and 6.8%, respectively. As such, EBF was 14.4%, and anemia in women of reproductive age was 15.9% [3, 4]. Since then, Mexico has made progress in reducing all forms of maternal and child malnutrition, but significant challenges remain. The ENSANUT 2022 recently published reveals staggering statistics and the distressing reality of maternal and child malnutrition in Mexico. The data reveals that in 2021, at the national level, 12.6% of children under five suffer from stunting and 1.5% from wasting, while 7.8% experience overweight; in 2012, the prevalence was 13.6%, 1.6%, and 9.7%, respectively [5]. However, these reductions are not statistically significant. The prevalence of anemia among women of childbearing age in Mexico shows an increasing trend from 11.6% in 2012 to 15.8% in 2022 [6, 7], signaling a nutritional deficiency often resulting in adverse long-lasting consequences on health for both mother and child and development for the child [7, 8]. Regarding EBF in children < 6 months, it rises from 14 to 33.6% [9, 10]. Although these numbers show an increasing trend, they continue far from the global goals (50% by 2025 and 70% by 2030). Otherwise, data from the Maternal and Child Observatory in Mexico (OMI, for its acronym in Spanish) reported that the incidence of LBW increased from 6.4% in 2017 to 7.1% in 2021 [11, 12].

These statistics reflect that maternal and child malnutrition remains a critical public health concern in Mexico and will only be able to meet one of the six nutrition targets (wasting) by 2025. It is still far from reaching those corresponding to stunting, low birth weight, anemia, and EBF by 2030, underscoring the urgent need to take immediate action to address the multifaceted challenges of all forms of maternal and child malnutrition.

Malnutrition has deeper structural drivers embedded in society that must be resolved to move forward toward meeting the targets. For example, in Mexico, 12.1% of the population has an income below the Extreme Poverty Line (monetary value of the food basket), representing 15.5 million people who do not have sufficient income to purchase the products of the basic food basket [13]. In addition, in the last four years, the number of Mexicans lacking access to health services has considerably increased from 16.2 to 39.1% between 2018 and 2022 [13]. The high rates of extreme poverty and the lack of access to health services coexist with other determinants that not only contribute to malnutrition but lead to worse health disparities and adverse health across the life course, such as food insecurity, the precarious nature of informal work, illiteracy, and the lack of robust social protection.

During the past decades, Mexico has implemented various strategies to address poverty and maternal and child malnutrition, such as the national conditional cash transfer program Prospera (formerly Progresa and Oportunidades), which was abolished in 2018 after 21 years of implementation. The “Estrategia Integral de Atención a la Nutrición (Comprehensive Strategy for Nutrition or EsIAN)- was a national strategy within Prospera that aimed to strengthen Prospera’s health and nutrition component by addressing the nutritional transition in Mexico and improving the health and nutrition of its beneficiaries, focusing on the first 1,000 days. Even though the EsIAN alone was insufficient to address the double burden of malnutrition in Mexico, from 2018 to the present, no program has been implemented to replace it and to attend to the most vulnerable groups, such as poor maternal and child populations at the national level [14].

The National Crusade Against Hunger was another program sponsored by the Mexican government from 2013 to 2018, aimed at reducing hunger and guaranteeing nutritious, adequate, and quality food; however, some factors, such as inadequate funding, bureaucratic obstacles, and the conflict of interest when incorporating companies such as Nestle or Pepsico as investors undermined the impact and hamper their effectiveness [15]. In 2022, an agreement was issued in the Official Gazette of the Federation (DOF) issuing the Health Care Model for Well-being (MAS-BIENESTAR) in which the “National Strategy of the first 1000 days” as a priority strategy [16]; however, it is essential to note that this strategy has not yet achieved nationwide coverage. Furthermore, the components that comprise it, its operation, and the efficient management of resources have yet to be precisely defined. Other efforts and actions are aimed at tackling other forms of malnutrition in Mexico, such as overweight and obesity. Some of them are policies to improve the food environment, such as the tax on sugar-sweetened beverages since 2014 or the front-of-package warning labeling system approved by the Mexican parliament in October 2019 [17, 18].

Despite concerted efforts, the prevailing national nutritional context demands a critical reassessment of the existing strategies and a deeper understanding of the structural and systemic barriers that impede progress. Furthermore, climate change and the emergence of sanitary emergencies such as that due to the COVID-19 pandemic significantly threaten food security and nutrition. As a result, Mexico faces a series of challenges ranging from droughts to storms, floods, and hurricanes. In October 2023, the city of Acapulco was affected by Hurricane Otis, leaving more than a third of its population injured (250,000 persons) [19]. These extreme weather events disrupt agricultural production, compromise nutritious food availability, and exacerbate marginalized and poorest communities’ challenges, further worsening malnutrition in all its forms.

The current and harsh situation in Mexico calls for urgent policy reforms addressing the root causes of malnutrition through multisector and multicomponent actions, with realistic and measurable targets, allocating resources strategically with a gender perspective, and forging a path toward sustainable progress in addressing malnutrition during preconception, pregnancy, infancy, and preschool age. In addition, policies and interventions must have an evaluation component for monitoring progress on reaching the established goals.

To achieve the global nutrition targets by 2030, addressing the profound structural barriers in Mexico is crucial. Mexico’s government must prioritize poverty reduction, reduce the marked inequalities, enhance the quality of nutritional care and healthcare infrastructure, and implement climate-resilient agricultural practices to address this pressing issue. Moreover, increasing investment in education and awareness campaigns is vital to promoting healthy dietary practices and improving nutritional outcomes. A multi-sectoral approach, strong political commitment, and sustained efforts from government agencies, civil society, and the private sector, with no conflict of interest, are essential to creating programs and policies promoting healthy lifestyles and creating healthy environments that make it possible to have healthy food and drink as a first choice and physically active life. Another essential aspect to consider will be ensuring monitoring progress and addressing emerging challenges.

By addressing these challenges head-on, Mexico can pave the way towards a healthier and more prosperous future for its citizens, ensuring the achievement of global targets in the long run; otherwise, the health of current and future generations will continue to be compromised and along with it the human, social and economic development of Mexico.

## Data Availability

Not applicable.

## Abbreviations

EBF: Exclusive breastfeeding
DOF: Official Gazette of the Federation
UNICEF: United Nations Children’s Fund
WHA: World Health Assembly
WHO: World Health Organization
